# Apelinergic system in the kidney: implications for diabetic kidney disease

**DOI:** 10.14814/phy2.13939

**Published:** 2018-12-10

**Authors:** Tilman Müller, Anastasia Z. Kalea, Alonso Marquez, Ivy Hsieh, Syed Haque, Minghao Ye, Jan Wysocki, Michael Bader, Daniel Batlle

**Affiliations:** ^1^ Department of Medicine Division of Nephrology and Hypertension Feinberg School of Medicine Northwestern University Chicago Illinois; ^2^ Charité‐Universitätsmedizin Berlin Berlin Germany; ^3^ Institute of Liver and Digestive Health University College London London UK; ^4^ Max Delbrück Center for Molecular Medicine Berlin Germany; ^5^ German Center for Cardiovascular Research (DZHK), partner site Berlin Berlin Germany; ^6^ Berlin Institute of Health (BIH) Berlin Germany; ^7^ University of Lübeck Lübeck Germany

**Keywords:** APJ, apelin, diabetic kidney disease, kidney

## Abstract

The bioactive peptides of the apelinergic system and its receptor APJ have been shown to play a protective role in experimental cardiovascular and diabetic kidney disease (DKD). Mechanisms of this renoprotective effect remain to be elucidated. In this study, we examined the localization of APJ within the normal kidney and its kidney expression in the *db/db* model of DKD. The effect of hyperglycemia and angiotensin II on APJ was examined in cultured podocytes. In the glomerulus, APJ colocalized with podocyte but not endothelial cell markers. In podocytes stimulated with *Pyr* ^1^Apelin‐13, a change in the phosphorylation status of the signaling proteins, AKT, ERK, and p70S6K, was observed with an increase 15 min after stimulation. Apelin‐13 decreased activity of Caspase‐3 in podocytes after high glucose treatment reflecting an antiapoptotic effect of APJ stimulation. In podocytes, APJ mRNA was downregulated in high glucose, when compared to normal glucose conditions and exposure to angiotensin II led to a further significant decrease in APJ mRNA. APJ and *preproapelin* mRNA levels in kidneys from *db/db* mice were markedly decreased along with decreased tubular APJ protein by western blotting and immunostaining when compared to *db/m* controls. In conclusion, the apelinergic system is decreased in kidneys from *db/db* mice. Within the glomerulus, APJ is mainly localized in podocytes and in this cell type its activation by Apelin‐13 abolishes the proapoptotic effect of high glucose, suggesting a potential therapeutic role of apelin and emerging agonists with extended half‐life for therapy of DKD.

## Introduction

APJ is a G protein‐coupled receptor consisting of seven transmembrane domains, that shares a 31% identical amino acid sequence with angiotensin AT1 receptor, but does not bind angiotensin II (Ang II) (Huang et al. 2018). Messenger RNA (mRNA) molecules for *preproapelin* and *APJ* are expressed in human tissues such as stomach, brain, heart, kidney, adipose tissue, lung, and in human endothelial cells (Hosoya et al. 2000; O'Carroll et al. 2000; Medhurst et al. 2003; Kleinz and Davenport 2004; Kleinz et al. 2005). The Apelin gene encodes an initially translated 77‐aa preproprotein, which is cleaved by proteases to shorter forms, such as Apelin‐36, ‐17, ‐13 or ‐the more resistant to degradation‐ pyroglutamate form of Apelin‐13 (*Pyr* ^1^Apelin‐13) (Medhurst et al. 2003; De Mota et al. 2004). The biological importance of Apelin as a peptide is suggested by its wide tissue distribution and the strict conservation of the last 13aa at the C‐terminus (65‐77aa) among all species studied (Tatemoto et al. 1998; Habata et al. 1999; Lee et al. 2000). In the cardiovascular system, both Apelin and APJ are present in cardiac myocytes and vascular smooth muscle cells (Kleinz and Davenport 2004; Hashimoto et al. 2006). The therapeutic potential of apelins in cardiovascular disease and preeclampsia is a topic of great interest (Kalea and Batlle 2010; Yamaleyeva et al. 2015, 2016; Zhe et al. 2016). Also of interest is a recent report that has shown that aging promotes and it is later characterized by a state of multiorgan apelinergic deficiency (Rai et al. 2017).

In the rat kidney APJ, mRNA expression has been reported (Hosoya et al. 2000; O'Carroll et al. 2000). APJ is highly expressed in the inner stripe (IS), intermediate in the outer stripe (OS) and in the inner medulla (IM) and, lowest in the cortex (Hus‐Citharel et al. 2008). O'Carroll et al. (2000) found that labeling in the kidney cortex corresponded to an APJ mRNA expression in 40% of the glomeruli, and suggested a role for this receptor in the regulation of blood flow. Studies focusing on the vasculature showed that the apelinergic system regulates Ang II ‐ AT1 receptor signaling (Susztak et al. 2006). It is possible that at the kidney level Apelin may also play a counterregulatory role on the actions of Ang II. In kidneys, studies on the role of Apelin and APJ in renal hemodynamics showed that Apelin increases medullary blood flow by inducing a vasodilatory effect in agreement with the opposing actions against Ang II regarding blood pressure (Ishida et al. 2004) and vascular tone (Gurzu et al. 2006; Zhong et al. 2007). Apelin has been shown to be protective against acute renal injury (Chen et al. 2015) and diabetic kidney disease (DKD) (Day et al. 2013). Day, Cavaglieri, and Feliers (Day et al. 2013) showed that Apelin‐13 reduces glomerular hypertrophy and inflammatory markers in the *OVE26* model of type 1 diabetes. Our study was aimed at determining the localization of the APJ receptor within the kidney glomerulus and examining its regulation by hyperglycemia and Ang II as well as signaling by apelin‐13 using cultured podocytes. Finally, the *db/db* mouse was used to examine the kidney expression of the apelinergic system in a model of type 2 diabetes.

## Materials and Methods

### Animal model and tissue preparation

Obese *db/db* mice (C57BLKS/JLepr) were used as a model of type 2 diabetes and their lean littermates (*db/m*) served as nondiabetic controls (Jackson Laboratory, Bar Harbor, ME). We used young (8 weeks of age) female *db/db* mice to study an early phase of diabetes (3–4 weeks of onset) and 24–32 weeks old female *db/db* mice which exhibit kidney lesions consistent with early diabetic nephropathy (Lee and Bressler 1981). The Institutional Animal Care and Use Committee approved all procedures. Kidneys and hearts from these mice were removed quickly, cut longitudinally, and half of kidney and heart sections were stored at −80°C for protein and RNA analysis. The remaining half of kidney sections were fixed with 10% buffered formalin phosphate (Fisher Scientific, Hanover Park, IL) overnight. After paraffin embedding, tissue sections (4 *μ*m) were deparaffinized in xylene and rehydrated through graded ethanol series before use.

### Kidney histology

For histological evaluation, kidneys were cut longitudinally and fixed with 10% buffered formalin phosphate (Fisher Scientific, Pittsburg, PA). Paraffin sections were stained with hematoxylin and eosin and periodic acid‐Schiff. Glomerular hypertrophy was quantified by measuring the glomerular tuft cross‐sectional area with a computer image analysis system (Image J, NIH). Glomerular hypertrophy was assessed by measuring the tuft area from glomeruli in which the vascular pole was evident (using 20 glomeruli per section). This was performed to reduce the possibility of including tangentially cut glomeruli (Soler et al. 2007). Glomerular images were obtained digitally using Tissue Gnostic Acquisition platform. Total glomerular area was traced, and calculated using Image J (NIH) analysis software. Mesangial matrix expansion and glomerular cellularity was graded by a masked renal pathologist using a semiquantitative score.

### Preparation of RNA and protein extracts

Kidney and heart sections were used to extract total RNA using TRIZOL^®^ (Thermo Fisher Scientific, Waltham, MA) following the manufacturer's instructions. Total RNA was quantified and tested for purity by optical density (OD) absorption ratio OD260 nm/OD280 nm with a spectrophotometer (GeneQuant Pro, Biochrom, Cambridge, United Kingdom). RNA was reverse transcribed using Reverse Transcription Kit (Thermo Fisher Scientific, Waltham, MA) following manufacturer's protocols and cDNA was stored at −20°C for further analysis. For protein extracts, kidney sections were washed with PBS over ice and protein was extracted using mammalian protein extraction buffer (MPER, Pierce) supplemented with a protease inhibitor cocktail (Sigma‐Aldrich, Saint Louis, MO) and PMSF, following manufacturer's instructions. Protein concentrations in protein homogenate supernatants were measured with the BCA assay method (Pierce, Rockford, IL). Supernatants were stored at −70°C and used for detection of proteins of interest.

### Reverse Transcription and RT‐qPCR

Constant amounts of 2.5 *μ*g of extracted kidney and heart RNA were reverse transcribed to synthesize complementary DNA (cDNA). Synthesis of cDNA was performed using Reverse Transcription Kit on a GenAmp PCR System 9700 (both Thermo Fisher Scientific, Waltham, MA) with standard cycling parameters. The mRNA expression for the genes of interest was quantified by RT‐qPCR on a 96‐well plate using a TaqMan Gene Expression Master Mix on a Step One Plus PCR System (both Thermo Fisher Scientific, Waltham, MA). PCR reactions were carried out using Assays‐on‐Demand™ Gene Expression Products (Thermo Fisher Scientific, Waltham, MA) following the suggested RT‐qPCR protocol for all investigated factors: denaturation for 10 min at 95°C, 40 cycles of a three segmented amplification and quantification program (denaturation for 10 sec at 95°C, annealing for 15 sec at the primer‐specific temperature (95°C), annealing/extension for 1 min at 60°C). Reactions were performed in duplicates. mRNA expression for *preproapelin* and *APJ* was normalized against glyceraldehyde‐3‐phosphate dehydrogenase (GAPDH) mRNA expression. GAPDH was chosen as internal control, since the levels were consistent among kidney and heart tissues as described in previous reports (Medhurst et al. 2003). Primers were ordered from Thermo Fisher (GADPH: Mm99999915_g1; APJ: Mm00442191_s1; Preproapelin: Mm00443562_m1).

### Western Blot

**Figure 1:**
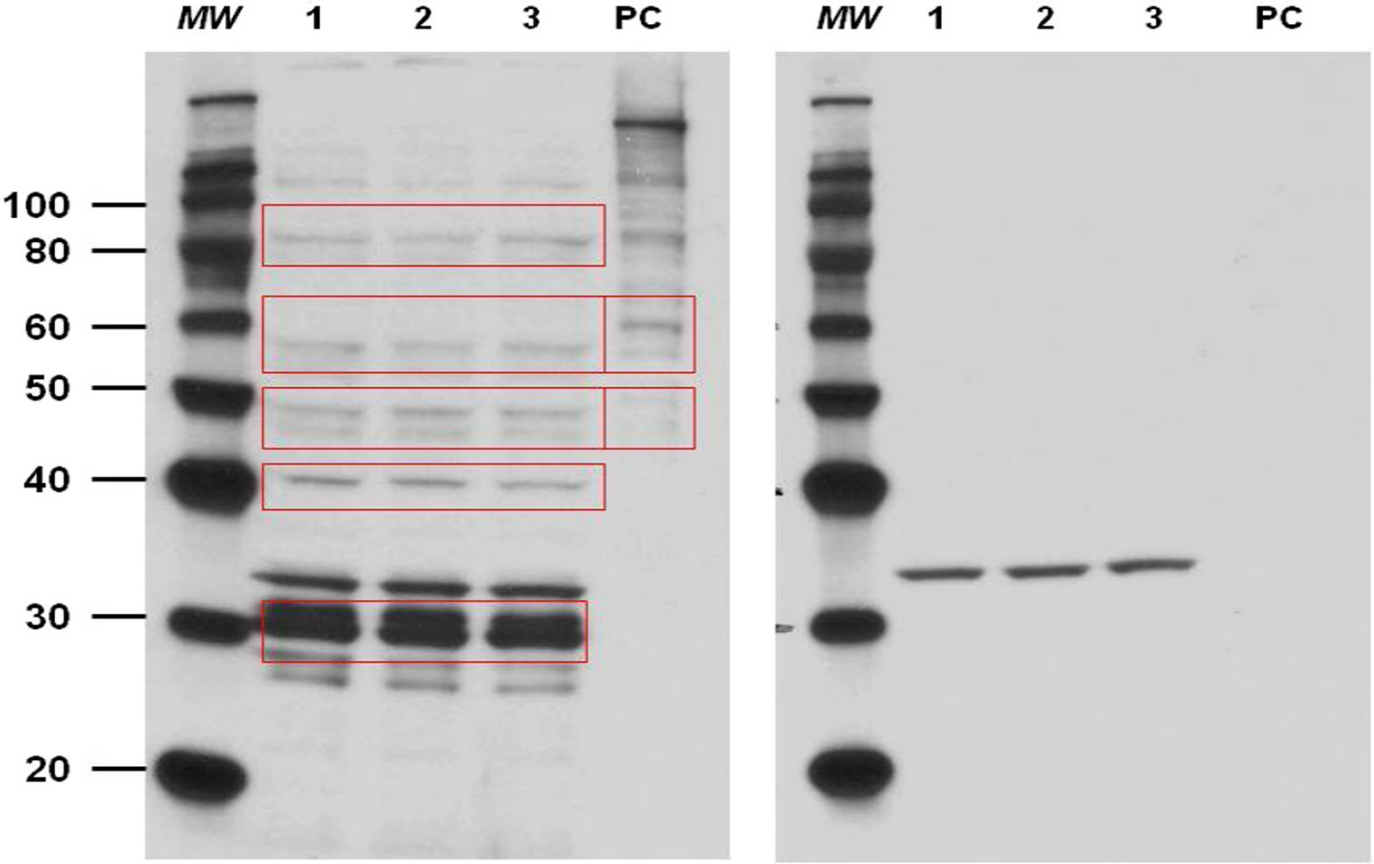
Immunoblot analysis for APJ in mouse kidneys (*n* = 3) without (left) and with (right) blocking peptide incubation. MW; molecular weight marker, PC; positive control, whole cell lysate from 293T‐transfected cells with human APJ, 1,2,3; lysates from three different mouse kidneys; red rectangular shapes highlight the main bands erased by the blocking peptide.

Protein expression for APJ was studied using protein extracts from mouse kidney lysates (~20 *μ*g), which were separated on 10% Bis‐Tris Novex precast gels (Thermo Fisher Scientific, Waltham, MA) with MOPS buffer, after denaturation in reducing sample buffer. Proteins were transferred to treated 0.4 *μ*m PVDF membranes (Millipore, Billerica, MA) which were blocked using 5% nonfat dry milk in 0.1% v/v Tween‐30 Tris‐buffered saline (TBS‐T), and later were immunoblotted using a primary antibody for APJ (goat polyclonal IgG 1:500, raised against a peptide mapping within a C‐terminal cytoplasmic domain of APJ, Santa Cruz Biotechnologies, Dallas, TX) and its corresponding HRP‐linked secondary antibody. The respective blocking peptide for APJ was used on the same membrane to verify the specificity of the bands detected in kidney samples. For further studies, we focused on the 42 kDa band, based on the reported molecular size of the monomeric APJ (Cai et al. 2017) and the uncertainty as to the identity of the extra bands which were also erased by the blocking peptide in kidney lysates (Fig. 1). A positive control with 293T whole cell lysate from cells transfected with human APJ was also used (Santa Cruz Biotechnologies, Dallas, TX) along with the kidney samples (Fig. 1).

In all experiments, detection of signal was performed using Amersham ECL Plus reagents, and films were developed and quantified by densitometry using *ImageJ* Software. In many cases, to confirm equal loading of total protein into each lane, membranes were stripped and probed for the control protein *β*‐tubulin (rabbit polyclonal IgG for *β*‐tubulin, Santa Cruz Biotechnologies, Dallas, TX).

### Immunohistochemistry

Kidney sections (4 *μ*m) were deparaffinized and rehydrated. Antigen retrieval was performed with a pressure cooker at 120°C in target retrieval solution (Agilent Technologies, Santa Clara, CA). Endogenous peroxidase activity was blocked with 3% hydrogen peroxide (Thermo Fisher Scientific, Waltham, MA). The primary antibodies for anti‐APJ (1:100; rabbit antibody, Neuromics, Edina, MN) and WT‐1 (1:400; rabbit antibody, Santa Cruz Biotechnology, Dallas, TX) were applied overnight and one slide in each set of experiments was incubated only with nonimmune serum to be used as negative control to evaluate specificity. Sections for APJ and WT‐1 staining were washed and incubated with goat anti‐rabbit IgG conjugated with peroxidase‐labeled polymer (Agilent Technologies, Santa Clara, CA). Peroxidase labeling was revealed using a liquid diaminobenzidine substrate‐chromagen system (Agilent Technologies, Santa Clara, CA). Sections were counterstained with hematoxylin (Sigma‐Aldrich, Saint Louis, MO) and dehydrated, mounted with Permount (Thermo Fisher Scientific, Waltham, MA), and cover‐slipped. For anti‐APJ staining, sections were examined and photographed with a Nikon Eclipse 50i microscope for semiquantitative and qualitative observations and comparisons. For assessment of the intensity of APJ kidney staining, a semiquantitative analysis of the immunoperoxidase stained sections was done based on fooling scale: 0‐ no staining; 1‐weak staining; 2‐strong staining. Sections were examined independently by two observers, who assessed staining intensity from each slide of either five viewing fields (for tubular staining) or 25 glomeruli (for glomerular tuft staining). A composite score was then generated for each mouse to compare APJ staining in *db/m* and *db/db* mice.

For podocyte count, two masked observers counted WT‐1 stained nuclei in 20 glomeruli from each kidney section.

### Confocal immunofluorescence microscopy

The paraffin‐embedded kidney sections (4 *μ*m) were deparaffinized and rehydrated. After antigen retrieval, sections were permeabilized with 0.5% Triton‐X100 in PBS for 5 min and blocked with 5% normal donkey serum in PBS for 1 h at room temperature in a humidified slide chamber. The sections were incubated with primary antibodies diluted in 5% donkey serum in PBS overnight at 4°C. The primary antibodies used for the immunofluorescence were anti‐APJ (1:200; rabbit antibody, Neuromics, Edina, MN) and one of the specific cell type markers. For the APJ antibody used for IF, there is no commercially available peptide immunogen. This anti‐APJ antibody, however, had been validated by the producer using APJ‐transfected COS‐7 cells (Neuromics, Data Sheet). Moreover, in a study by Farkasfalvi et al. (2007) in addition to the antibody, we used a second APJ antibody directed against a different region of the receptor to ensure robust results. They determined that both antibodies showed the same staining pattern and hence both were equally specific. As podocyte markers, we used antinephrin (1:100; Santa Cruz Biotechnologies, Dallas, TX), which localizes specifically in the slit diaphragm (Zhang et al. 2004); an antibody against synaptopodin (1:100; Santa Cruz Biotechnologies, Dallas, TX), which is an actin‐associated protein in the podocyte foot process (Thomas et al. 1994); antipodocin (diluted 1:100; Santa Cruz Biotechnologies, Dallas, TX), which is specific for the basal pole of podocyte along the glomerular basement membrane (Roselli et al. 2002) as well as anti‐WT‐1 (diluted 1:100; Santa Cruz Biotechnologies, Dallas, TX) and DAPI (Santa Cruz Biotechnologies, Dallas, TX), for specific podocyte nuclear staining. Platelet‐endothelial cell adhesion molecule (PECAM‐1) antibody (1:100; Santa Cruz Biotechnologies, Dallas, TX) was used as an endothelial cell marker of glomerular endothelial cells and *α*‐smooth muscle actin antibody (diluted 1:200; Sigma‐Aldrich, Saint Louis, MO) was used to stain vascular smooth muscle (Ye et al. 2006). Desmin, a gift from J Meiner, was used as a marker of mesangial cells (Lin et al. 2018). Antibodies against ACE2 (diluted 1:200, affinity purified (Ye et al. 2006)), ACE (Ye et al. 2006) and aquaporin 2 (diluted 1:200, Santa Cruz Biotechnology, Dallas, TX) were used for colocalization studies within proximal and collecting tubules. Sections were washed with PBS‐T three times and then incubated for 45 min with one of the respective fluorescent secondary antibodies (diluted 1:200; Alexa Fluor 488 donkey anti‐rat, Alexa Fluor 555 donkey anti‐rabbit, Alexa Fluor 647 donkey anti‐goat, and Alexa Fluor 647 donkey anti‐mouse IgG; Molecular Probes, Eugene, OR). Sections were washed three times and cover slips were placed carefully on top of one drop of Prolong Gold antifade reagent (Molecular Probes, Eugene, OR), and sealed with nail polish. Negative controls for immunofluorescence staining were performed by substitution of nonimmune serum for the primary antibodies in adjacent sections (Lambrecht et al. 2006). All slides were visualized with a Zeiss LSM 510 confocal microscope (Carl Zeiss, Jena, Germany).

### ELISA measurements in urine and plasma samples

Plasma and urine samples of *db/m* and *db/db* mice were tested for Apelin concentration (pg/ml) using a fluorescent enzyme‐linked immunosorbent assay (ELISA) directed against the C‐terminus of Apelin‐12 (Phoenix Pharmaceuticals, Belmont, CA) following the manufacturer's protocol. The used kit is designed to detect the C‐terminus of other active forms of Apelin, including Apelin‐36 and Apelin‐13 (sensitivity: 15.8 pg/mL).

In urine samples, we also measured Angiotensinogen (AOG) by a quantitative solid‐phase sandwich ELISA (IBL‐America, Minneapolis, MN, sensitivity: 30 pg/mL) as well as Ang II using an EIA Kit from Cayman Chemical (Ann Arbor, MN, sensitivity: 1 pg/mL). This assay had less than 0.001% cross‐reactivity with Ang (1–7) and 4% cross‐reactivity with Ang I(1–10). Creatinine concentration was assessed using the Jaffe method (Creatinine Companion, Exocell, Philadelphia, PA) and used for correction of urinary values of Apelin‐12, AOG and Ang II.

For urinary peptide measurements, an aliquot of 100 *μ*l of freshly collected urine was transferred into tubes kept on ice at 4°C containing 10X concentrated cocktail of peptidase inhibitors: 25 mmol/L EDTA, 0.44 mmol/L o‐phenanthroline, 1 mM chloromercuribenzoic acid (PCMB), and 120 mmol/L pepstatin A in PBS mixed thoroughly. The urines with inhibitors were then stored at −80°C until the extraction. Angiotensin peptides were extracted from urines using reverse‐phase phenyl silica columns (Thermo Scientific cat. no. 60108‐386, 100 mg) according to the manufacturer's instructions. Urinary angiotensin II and Apelin levels were measured using EIA kits.

### Cell culture

Conditionally immortalized mouse podocytes generated by Dr. Peter Mundel (Massachusetts General Hospital, Boston, MA) were cultured as previously described (Chen et al. 2004; Lee et al. 2006; Shankland et al. 2007). For the studies, passages 23‐28 were used. The cultured cells exhibited epithelial morphology and were characterized as podocytes by detection of the podocyte‐specific markers: podocin, synaptopodin, and nephrin, by immunofluorescence staining (see results). The cells were allowed to differentiate for at least 2 weeks at 37°C without *γ*‐interferon in DMEM (Gibco Laboratories) containing 5.5 mmol/L glucose and with 5% heat‐inactivated FBS (Gibco Laboratories). The medium was refreshed every 3 days, and the cells were subcultured upon confluence. These cells were then used for apoptosis and cell signaling studies. For qPCR studies, podocytes (Probetex, San Antonio, TX) were starved 24 h before measurements at 37°C in a 5% CO_2_ atmosphere in 5.5 mmol/L glucose DMEM (low glucose condition, LG) or 25 mmol/L DMEM (high glucose condition, HG) without FBS. In a second approach, podocytes were starved 24 h before measurements under high glucose condition with or without addition of 0.1 *μ*mol/L of Ang II. In this setting, media were exchanged once after 12 h. The medium was aspirated after 24 h and the dishes were washed with 4 mL of ice cold PBS followed by RNA extraction by TRIZOL (as described above). For cell culture studies, the PCR was performed using a SYBR Green qPCR Master Mix Kit (Thermo Fischer Scientific, Waltham, MA) by applying standard cycling parameters and using primers (IDT, Coralville, IA) specifically designed for this type of qPCR (See below).

Primers for RT‐qPCR

**Table:** 

Primer	Sequence 5′‐3′	Accession Number
APJ F	TTT GGA GCA GCC GAG AAA	AB033170.1
APJ R	GTC AAA CTC CCG GTA GGT ATA AG	AB033170.1
Apelin F	TCC AGA TGG GAA AGG GCT	AB023495.1
Apelin R	CTG TCT GCG AAA TTT CCT CCT	AB023495.1
GADPH F	ACT CCC ATT CTT CCA CCT TTG	AB017801.1
GADPH R	CCC TGT TGC TGT AGC CAT ATT	AB017801.1

### Cell signaling assays

Activation of cell signaling proteins was evaluated by western blot using phosphorylation specific antibodies on podocytes stimulated with *Pyr* ^1^‐Apelin‐13. Podocytes were seeded in 100 mm cell culture plates (~10^6^ cells/plate). Subconfluent cells were serum‐deprived overnight and then stimulated with *Pyr* ^1^‐Apelin‐13 (Bachem, Bubendorf BL, Switzerland) for 1, 5, 15, and 60 min. Protein was then extracted using a mammalian protein extraction reagent (MPER, Pierce) supplemented with protease (Sigma‐Aldrich, Saint Louis, MO) and phosphatase (Roche, Basel, Switzerland) inhibitor cocktails. Lysates containing equal quantities of total protein were separated in 10% Bis‐Tris Novex precast gels and proteins were transferred to nitrocellulose membranes (Millipore, Billerica, MA). After blocking, membranes were incubated with primary antibodies for phospho‐specific and total AKT, p70S6K, and ERK (Cell Signaling) and their corresponding HRP‐linked secondary antibodies. Detection of signal was performed using Amersham ECL Plus reagents, and films were developed and quantified by densitometry using ImageJ Software.

### Caspase‐3 Assay

The assay was performed using a kit from Cayman Chemical, Ann Arbor, MI Conditionally immortalized podocytes were serum starved 24 hr before the experiment with RPMI 1640 and 0.2% fetal bovine serum and seeded onto a 96‐well plate. Apelin‐13 (100 nmol) was diluted in RPMI medium with high glucose (25 mM) or normal glucose (11.1 mmol/L) content and 100 *μ*L of the solution was put to the cells. Cells were incubated at 37°C overnight in a 5% CO_2_ atmosphere. The plate was centrifuged at 800 g for 5 min and medium was aspirated; 200 *μ*L of Caspase‐3 assay buffer was added to each well and the plate was centrifuged again at 800 g for 5 min. After adding 100 *μ*L of cell‐based assay lysis buffer to each well, the plate was placed on an orbital shaker for 30 min at room temperature. The plate was then centrifuged for 10 min at 800 g. The supernatant of each well (90 *μ*L) was then transferred to a corresponding well on a black 96 well plate and 10 *μ*L of Caspase‐3 Assay Buffer or 10 *μ*L of Caspase‐3 inhibitor solution was added. Finally, 100 *μ*L of Caspase‐3 substrate solution was pipetted in each well and the plate was incubated for 30 min at 37°C. Activity was measured at 485 nm excitation and 535 nm emission using a microplate fluorescence reader FLx800 (Biotek, Winooski, VT).

### Effect of AT1‐receptor blockade on kidney *APJ* and *preproapelin* mRNA levels

Telmisartan, a specific Ang II receptor antagonist, was given to *db/db* mice for 11 weeks starting at the age of 13 weeks. Mice were assigned to drink either tap water (*n *=* *6) or tap water with telmisartan (Boehringer Ingelheim, Ingelheim am Rhein, Germany) at a dose of 2 mg*kg^−1^*day^−1^ (*n *=* *6). For telmisartan administration, mice were weighed and the daily fluid intake per mouse was recorded to estimate the concentration of the compound needed to be added to the drinking water.

### Statistical analysis

In all experiments, unless otherwise indicated, data were reported as mean ± SEM with at least three replicates per group. Pairwise comparisons were performed using two‐tailed *t* test (for normally distributed data) or Mann–Whitney test (for not normally distributed data) and a *P* value < 0.05 was considered significant.

## Results

### *APJ* mRNA and *Preproapelin* mRNA in mouse kidney

The relative amount of *APJ* mRNA and *preproapelin* mRNA were evaluated using RT‐qPCR in kidneys from nondiabetic *db/m* mice (*C57BLKS/JLepr*). For comparison, the mRNA levels are reported in relation to those observed in heart tissue where expression of the *APJ* receptor and *preproapelin* are known to be abundant (Medhurst et al., 2003). *APJ* receptor mRNA levels in the kidney were clearly detectable but only at about 1/8 of the heart mRNA (1.0 ± 0.7 vs. 7.7 ± 1.9 AU). By contrast, *preproapelin* mRNA levels were not significantly different between kidney and heart (1.0 ± 0.1 vs. 1.6 ± 0.9 AU).

### Localization of APJ protein in mouse kidney

**Figure 2:**
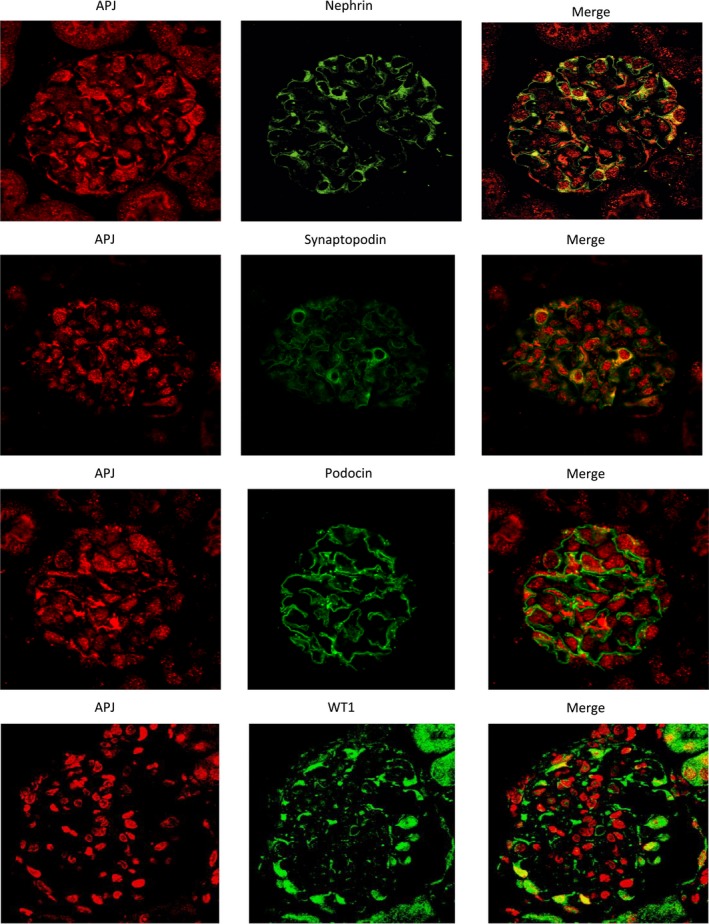
Immunofluorescence staining of APJ (red; left panels) and the podocyte markers nephrin (green; upper panel), synaptopodin (green, second panel from the top) and podocin (green, third panel from the top) in a glomerulus from mouse kidney. APJ shows strong colocalization with nephrin (right upper panel) and synaptopodin, (right second panel) and weaker with podocin (right third panel). The bottom panels show staining of APJ (red; left) and the podocyte nuclear marker WT1 (green; middle). Double‐staining reveals APJ colocalization within WT1‐positive nuclei in some podocytes (right).

To examine which kidney cell types express APJ immunofluorescent stained sections were evaluated by confocal microscopy in nondiabetic *db/m* mice (*C57BLKS/JLepr*). To localize APJ within the glomerulus, we utilized markers of epithelial glomerular cells (podocytes), mesangial and endothelial glomerular cells as previously described (Ye et al. 2006). Strong colocalization was found between APJ and nephrin, a podocyte marker (Fig. 2, top panel). APJ also colocalized with the podocyte marker synaptopodin, albeit not as strongly as nephrin, and weakly with podocin, another podocyte maker (Fig. 2, middle panels). Double‐staining with the nuclear marker WT1, also revealed some cells with APJ colocalization (Fig. 2, bottom panel).

**Figure 3:**
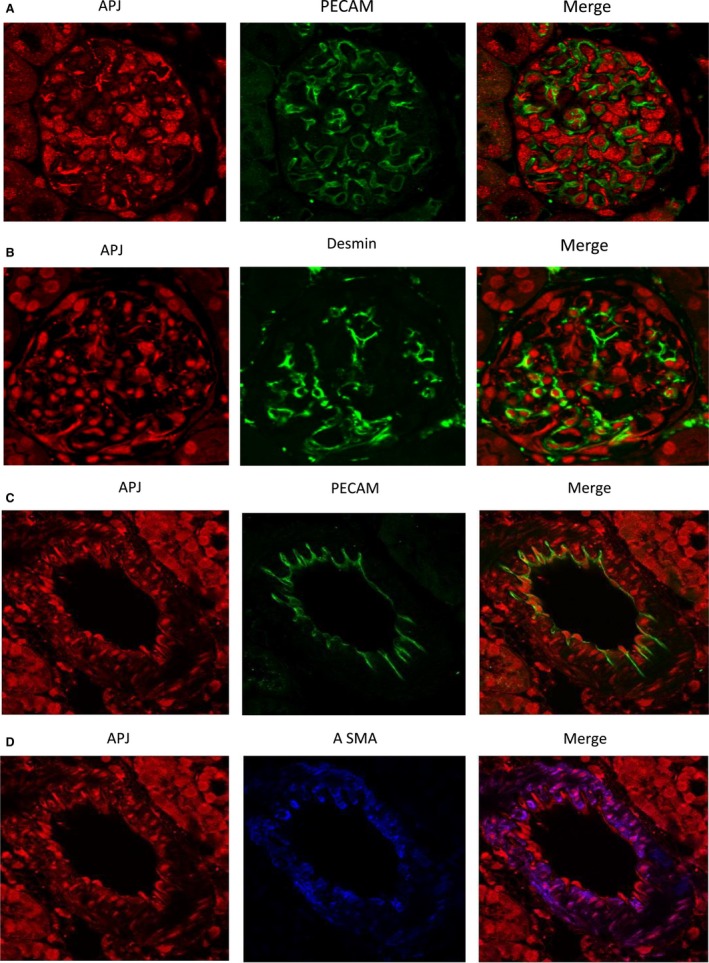
Immunofluorescence staining of APJ (red; left) and the endothelial cell marker, the platelet‐endothelial cell adhesion molecule, PECAM‐1 (green; middle; A) and desmin (green, middle; B) in a kidney glomerulus. APJ shows no colocalization with PECAM‐1 (right, A) and little with desmin (yellow; right, B). Double immunofluorescence staining of APJ (red; left, C) and the endothelial cell marker platelet‐endothelial cell adhesion molecule PECAM‐1 (green; middle; C) and the vascular smooth muscle marker, the *α*‐smooth muscle actin, *α*‐SMA (blue; middle; D) in a renal vessel from mouse kidney. APJ shows no colocalization with PECAM‐1 (right), but colocalizes strongly with *α*‐SMA (pink; right).

Staining of kidney glomeruli for APJ and PECAM‐1 was strictly separated (Fig. 3 panel A), indicating that APJ is not present in glomerular endothelial cells. Likewise, APJ showed little colocalization with the mesangial marker Desmin (Fig. 3, panel B). Further, no colocalization with the endothelial cell marker PECAM‐1 was found in renal arteries (Fig. 3, panel C). This is in concordance with absence of APJ in endothelial glomerular cells.

In renal arteries, APJ receptor colocalized with the smooth muscle marker *α*‐SMA reflecting its presence in the intima/tunica media as previously described (Pereira et al. 2013), but there are also areas with the vessel wall where no colocalization was seen (Fig. 3, panel D).

**Figure 4:**
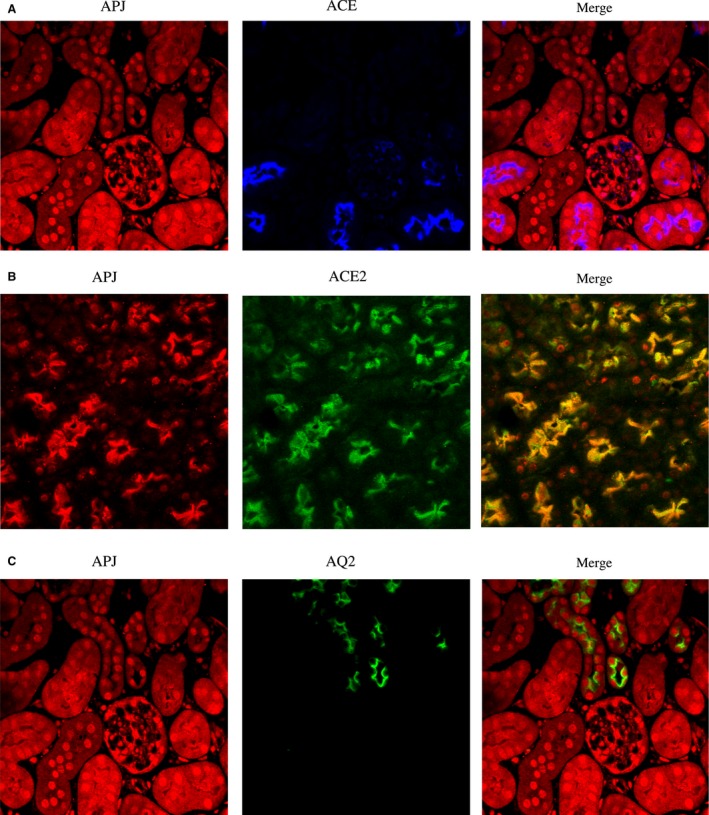
Immunofluorescence double‐staining of APJ (red; left) with ACE (blue; middle; A) and ACE2 (green; middle; B) in proximal tubule (A and B) as well as with aquaporin2 (green; middle; C) in collecting tubule (C). Images on the right are the digital overlay images (pink or yellow) for each respective row. APJ shows strong colocalization with ACE and ACE2 throughout the proximal tubule (pink; right panel A and yellow; right panel B). APJ is also localized in the collecting tubule, but there is very weak colocalization with aquaporin2 (panel C, right).

To localize APJ within tubules, we used ACE2 and ACE as markers for proximal tubules (Ye et al. 2006) and aquaporin‐2 for principal cells of the collecting tubules (Fig. 4). APJ colocalized with ACE and ACE2 (Fig. 4, panels A and B respectively) showing its presence throughout the proximal tubule. APJ colocalized but weakly with aquaporin‐2 (Fig. 4C).

### Studies in cultured podocytes

**Figure 5:**
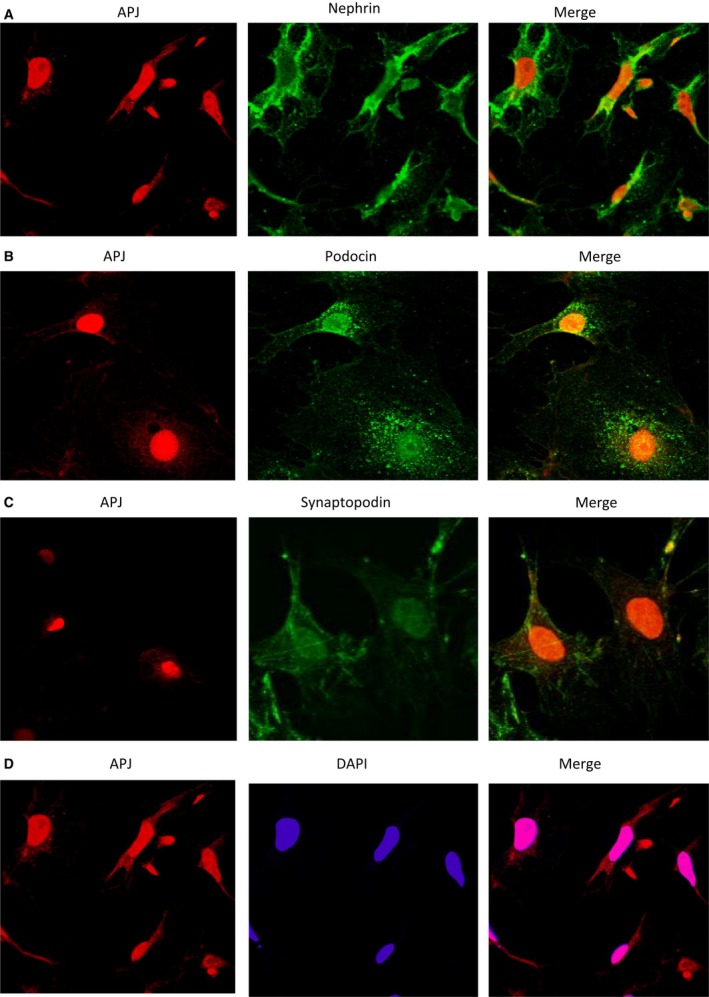
The podocytes were triple‐label immunostained with antibodies directed against APJ (red; left panels) and the podocyte markers nephrin, podocin, and synaptopodin (green A through C; middle) and DAPI as a nuclear counterstain (blue, middle, D). Images on the right are the digital overlay images (yellow or pink) for each respective row. Double‐staining shows colocalization of APJ with all three podocyte markers. APJ colocalizes with a nuclear marker DAPI, but is also found outside of the nucleus. (right, panel D)

Staining of cultured podocyte cells showed colocalization of APJ with podocyte markers (nephrin, synaptopodin and podocin) (Fig. 5). Nuclear presence of APJ was suggested by double‐staining with DAPI, a nuclear marker. APJ staining was also found outside the nucleus (Fig. 5).

#### Apelin signaling in cultured podocytes exposed to apelin‐13

After showing expression of APJ in podocytes, we examined whether there is a functional role of the Apelin system in this cell type. The cellular response to Apelin‐13 stimulation was determined by measuring the phosphorylation status of intracellular signaling proteins in cultured cells. Cells were stimulated with 100 nM *Pyr* ^1^Apelin‐13. The ratio of phosphorylated protein to total protein was determined for Akt, p70S6K, and ERK.

**Figure 6:**
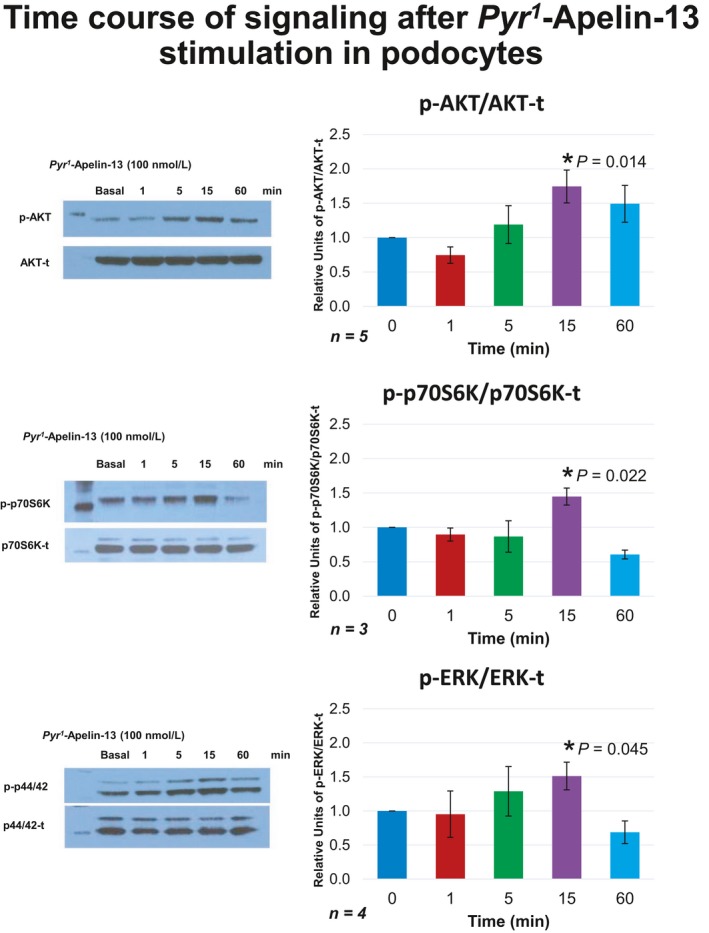
Time course of phosphorylation for AKT, (upper) p70S6K (middle) and ERK (lower) after stimulation with *Pyr* ^1^Apelin‐13 in cultured podocytes. Significant transient increases were observed in all three proteins 15 min after stimulation; suffix t – total; prefix p‐ phosphorylated form.

In podocytes, a significant transient increase in phosphorylation was seen for all three proteins (Akt, p70S6K, and ERK) 15 min after stimulation (Fig. 6). After 60 min, the increase subsided completely for ERK and p70S6K and partially for AKT, showing a dynamic system of Apelin signaling activity and its deactivation, as previously shown at similar time points in other cell types (Masri et al. 2004, 2006).

#### Effect of Apelin‐13 on Caspase‐3 activity

**Figure 7:**
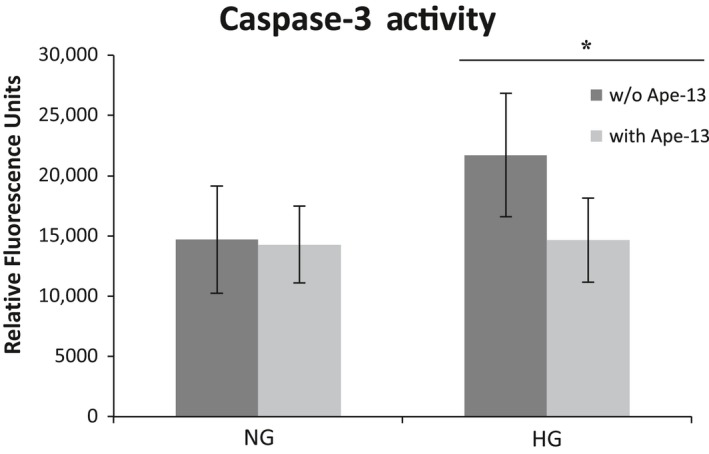
Caspase‐3 activity measured in cultured podocytes in normal glucose (NG) or high glucose medium (HG) with apelin‐13 (light gray, with Ape‐13) or without addition of apelin‐13 (dark gray, w/o Ape‐13). Caspase‐3 activity was significantly increased in the high glucose environment. Addition of apelin‐13 to cells kept in HG medium decreased activity levels down to levels measured in the NG environment. *P < 0.05, n of 7 experiments per group

Akt and ERK, the two signaling pathways that we found being activated by *Pyr* ^1^Apelin‐13, have been implicated in Apelin receptor‐mediated suppression of apoptosis (Liu et al. 2017). We therefore examined whether Apelin‐13 exerts an antiapoptotic effect in cultured podocytes under two glucose concentrations (11.1 mmol/L and, 25 mmol/L). Caspase‐3 activity was significantly higher with 25 than 11.1 mmol/L glucose conditions (21722 ± 5142 vs. 14685 ± 4438 RFU/*μ*g, *P *<* *0.05, *n *=* *7) (Fig. 7). Caspase‐3 activity in podocytes with added Apelin‐13 did not differ from activity measured without addition of Apelin‐13 (14285 ± 3189 vs. 14685 ± 4438 RFU/*μ*g, *P < 0.05*,* n *=* *7) (Fig. 7). Under the higher glucose concentration however, addition of Apelin‐13 markedly decreased Caspase‐3 activity (14655 ± 3517 vs. 21722 ± 5142 RFU/*μ*g, *P *<* *0.05, *n *=* *7). These findings show that in cultured podocytes grown in high glucose conditions, stimulation of APJ by Apelin‐13 reduces the proapoptotic effect of excessive glucose.

#### Effect of high glucose environment and Ang II on *APJ* and *preproapelin* mRNA expression

**Figure 8:**
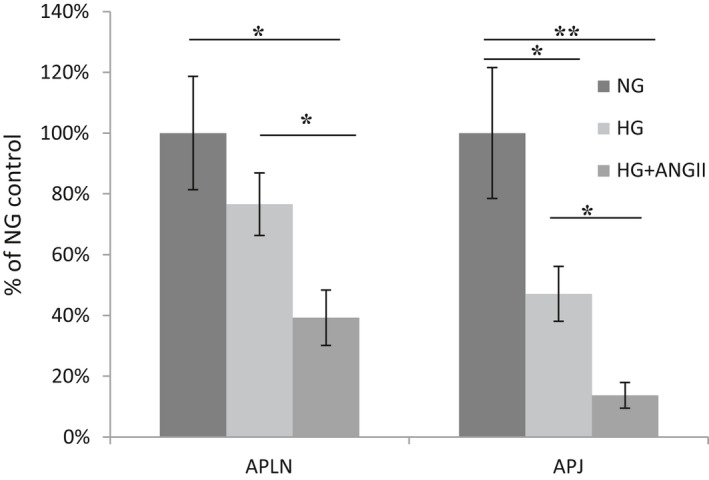
Relative mRNA levels of *preproapelin* (APLN, left) and *APJ* (right) in cultured podocytes in normal glucose medium (NG), high glucose medium (HG) or high glucose medium with 0.1 *μ*mol/L of Ang II (HG+ANGII). *APJ* mRNA was significantly decreased by HG, whereas *preproapelin* decreased but not significantly. Both *APJ* and *preproapelin* decreased significantly under HG with addition of Ang II; *P < 0.05; **P < 0.01.

RT‐qPCR revealed that a HG environment by itself significantly decreased APJ mRNA, 1.19 ± 0.26 versus 0.56 ± 0.11 (Fig. 8). *Preproapelin* mRNA also decreased but not significantly, 1.30 ± 0.24 versus 0.99 ± 0.13, *P* > 0.05 (Fig. 8).

Exposure to 0.1 *μ*mol/L Ang II for 24 h significantly decreased the expression of both APJ and *Preproapelin* mRNA under high glucose conditions (APJ: 0.56 ± 0.11 vs. 0.16 ± 0.05, *P* < 0.05; *Preproapelin*: 0.99 ± 0.13 vs. 0.51 ± 0.12, *P* < 0.05). The decrease in mRNA with addition of Ang II was higher than that achieved by high glucose alone for both preproapelin mRNA and APJ mRNA (Fig. 8).

### Studies in *db/db* mice and nondiabetic *db/m* controls

#### General and light microscopy findings

These studies were performed in mice of 24–32 weeks of age. Blood sugar of *db/db* mice at this timepoint was significantly increased as compared to the *db/m* model. Urinary albumin in *db/db* mice was significantly increased in comparison to nondiabetic *db/m* controls (Table 1). Body weight and kidney weight were increased in *db/db* mice, but the ratio of kidneys/body weight was decreased in *db/db* mice as compared to *db/m* controls, reflecting the profound obesity of the *db/db* animals.

**Table 1:** Characteristics of *db/m* and *db/db* mice at 24–32 weeks of age

	*db/m*	*db/db*
Blood glucose (mmol/L)	163 ± 9	530 ± 41
Body weight (g)	27.8 ± 0.5	49.5 ± 2.0*
Kidney weight (mg)	154 ± 9	206 ± 6*
Ratio kidneys/body weight (mg/g)	5.6 ± 0.4	4.2 ± 0.2*
Albumin/Creatinine ratio (μg/mg)	7.3 ± 2.0 (5)	613.3 ± 366.0* (5)
Mesangial matrix expansion	0.60 ± 0.24 (5)	2.20 ± 0.20* (5)
Glomerular cellularity	0.6 ± 0.24 (5)	1.8 ± 0.20* (5)
Glomerular tuft area (μm^2^)	2804.2± 111 (5)	3931.5 ± 252.6* (5)
Podocyte count (per glomerulus)	13.1 ± 0.3 (8)	10.0 ± 0.4* (8)

*db/m* control mice; *db/db* diabetic mice (*n* = 7 and *n* = 6 respectively, unless stated otherwise); Data are expressed as means ± SE.

* *P < 0.05* *db/m* versus *db/db* mice by Mann–Whitney test; Optical histology: mesangial matrix expansion and glomerular cellularity, were evaluated on H&E and PAS sections by a renal pathologist. Podocyte count was performed by counting WT‐1 stained nuclei in a blinded fashion by two independent investigators.

#### APJ mRNA, protein abundance, and immunohistochemistry in *db/db* mice

**Figure 9:**
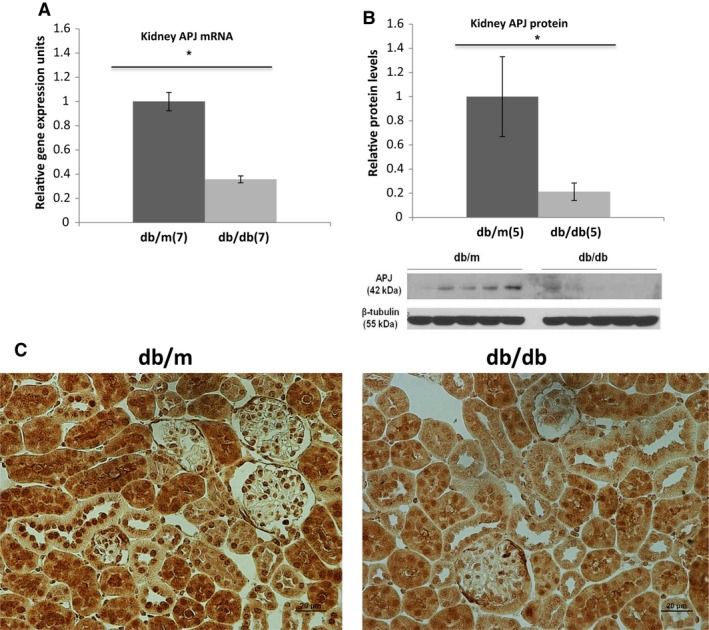
mRNA (A) and protein levels of APJ receptor by Western Blot (B) in whole kidney extracts of *db/m* and *db/db* mice. APJ receptor protein (42 kD) was normalized to the mouse *β*‐tubulin (55 kD) (B). APJ mRNA and protein levels are significantly decreased in *db/db* mice. The bar graphs represent mean±SEM values; *, *P* < 0.05 versus *db/m* mice. Immunohistochemistry (C) of kidney sections from *db/m* (left) and *db/db* (right). APJ staining (dark brown) is seen in tubules and to lesser extent in glomeruli. Staining was stronger in *db/m* as compared to *db/db* mice.

APJ mRNA levels were first measured in diabetic *db/db* mice in whole kidney extracts by RT‐ qPCR. Compared to *db/m* controls of the same age (8 weeks), APJ mRNA levels were markedly decreased in *db/db* mice (1.0 ± 0.076 vs. ± 0.36 ± 0.029, *n *=* *7, *P* < 0.01) (Fig. 9A). By Western blotting, the relative protein abundance of APJ was also markedly decreased in diabetic *db/db* mice as compared to *db/m* controls (Fig. 9B).

By immunohistochemistry, tubular APJ was also decreased in *db/db* mice compared to nondiabetic *db/m* controls (1.2 ± 0.1 vs. 1.6 ± 0.1, *P* < 0.05) (Fig. 9C). APJ stained weaker in glomerular tufts and a significant difference could not be found between *db/db* and *db/m* (1.77 ± 0.03 vs. 1.82 ± 0.03, respectively *P* = 0.2).

#### *Preproapelin* mRNA in the kidney and plasma apelin levels

When compared to *db/m* controls, mRNA levels of *preproapelin* were markedly decreased in kidneys from *db/db* mice (0.12 ± 0.016 vs. 1.0 ± 0.13 AU, *n *=* *8, *P* < 0.01).

Apelin‐12 was increased in plasma from *db/db* mice when compared to *db/m* controls (1.241 ± 0.175 vs. 0.786 ± 0.072 ng/ml, *n *=* *6, *P* < 0.05). Apelin‐12 in the urine from *db/db* mice was also increased when compared to *db/m* (1115.0 ± 323 vs. 504.4 ± 319 pg/mg creatinine, *n* = 12 for *db/db* and *n* = 8 for *db/m*), although this difference did not reach statistical significance (*P* = 0.054).

#### Urine Angiotensinogen and Ang II in *db/db* mice

In *db/db* mice of 8 weeks of age, urine Ang II (normalized by urine creatinine) was significantly higher when compared with *db/m* (203 ± 54 vs. 45 ± 13 vs. pg/mg creatinine, *P* < 0.05). Angiotensinogen (AOG) concentration normalized by creatinine was also significantly higher in *db/db* mice than in *db/m* controls (41.2 ± 9.7 vs. 5.9 ± 1.7 ng/mg creatinine, *P* < 0.05). Both may be attributed to an activation of the local kidney renin–angiotensin system in diabetic mouse models (Wysocki et al. 2017a). Albumin/creatinine ratio was significantly higher in *db/db* than in *db/m* controls (303.4 ± 32.1, *n *=* *25 vs. 87.3 ± 4.3 *μ*g/mg, *n *=* *15 respectively, *P *<* *0.01).

### Effect of Telmisartan on APJ in *db/db* kidney

**Figure 10:**
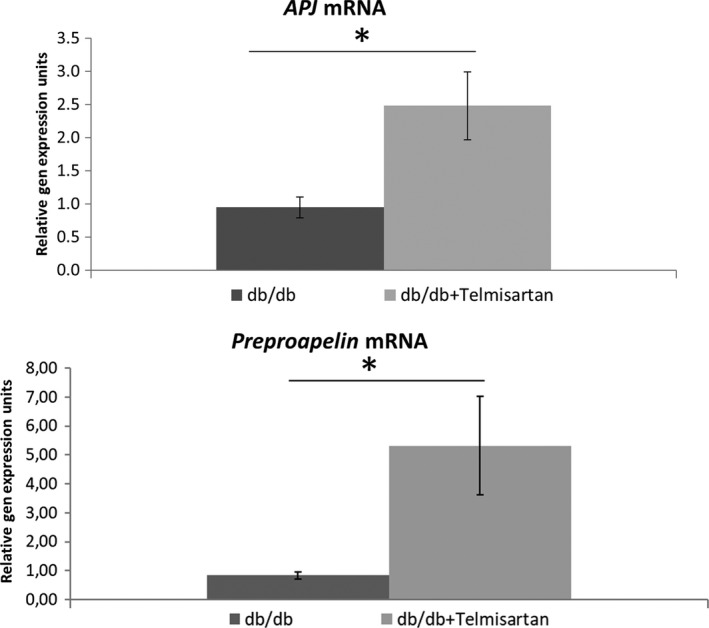
mRNA of *APJ* and *Preproapelin* measured by RT PCR in kidney lysates from untreated *db/db* mice and *db/db* mice given telmisartan, a specific AT1 blocker for 11 weeks (*n* = 6 per group); *P < 0.05.

Kidney *APJ* mRNA expression in *db/db* mice treated with telmisartan for 11 weeks was significantly higher as compared to untreated *db/db* mice (2.48 ± 0.51 vs. 0.95 ± 0.16 AU, *n *=* *6 both groups, *P *<* *0.01) (Fig. 10A). Kidney *preproapelin* mRNA was also significantly increased in kidneys from mice treated with telmisartan (5.41 ± 1.51 vs. 0.83 ± 0.11 AU, *n* = 6 both groups, *P *<* *0.05) (Fig. 10).

## Discussion

In this report, we examined the distribution of APJ in the kidney and demonstrated that within the glomerulus the receptor is localized in epithelial glomerular cells (podocytes) but not in glomerular endothelial cells and only very weakly in mesangial cells. Further, APJ was also abundantly present in proximal tubules, and renal vasculature, as previously described (O'Carroll et al. 2000). In diabetic *db/db* mice, we found that the overall expression of APJ was depressed at the mRNA level and also at the protein level both by Western Blot and by immunohistochemistry.

In cultured podocytes, Apelin‐13 stimulated APJ triggering the activation of Akt, Erk, and p70S6K pathways. The pattern of activation by apelin was brief and transient as previously described in other cell types, such as smooth muscle cells, neurons, adipocytes, and osteoblasts (Chaves‐Almagro et al. 2015). The Akt pathway has been described to activate endothelial nitric oxide synthase (eNOS), which is responsible for the vasodilatory effect of Apelin in vessels (Jia et al. 2007; Japp et al. 2008). Interestingly, within the podocytes the expression pattern was both nuclear as well as in the plasma membrane, as expected for a G protein‐coupled receptor (Lee et al. 2004).

We also found that apoptosis through activation of Caspase‐3 was increased in podocytes when they were exposed to a high glucose environment, confirming the data reported by Langer et al. (2016). We used Caspase‐3 activity as a marker of podocyte apoptosis, which is considered an early manifestation of diabetic lesions in kidneys from mouse models, such as the *db/db* (Susztak et al. 2006). Here, we show for the first time that stimulation by Apelin‐13 leads to a reduction of the otherwise elevated Caspase‐3 activity in high glucose environment in podocytes (Fig. 7). This decrease in Caspase‐3 activity in podocytes by Apelin‐13 is in accordance with similar antiapoptotic effects of the peptide in human and mouse osteoblasts (Tang et al. 2007; Xie et al. 2007), rat cardiomyocytes (Zhang et al. 2009), and rat brain tissue (Khaksari et al. 2012). Others have shown that Apelin affects cell survival by directly suppressing apoptosis (Liu et al. 2017). Together with activation of antioxidant enzymes like catalase (Nishida and Hamaoka 2013), reduction of Caspase‐3 activity may partially account for the renoprotective effect of the apelinergic system in DKD which has been previously reported by Day, Cavaglieri, and Feliers (Day et al. 2013). Importantly, in this study administration of Apelin‐13 to diabetic mice ameliorated DKD and upregulated kidney APJ (Day et al. 2013). Taking into account the localization of APJ in podocytes and Caspase‐3 suppression by Apelin‐13 in these cells, it is likely that stimulation of a podocyte apelinergic system accounts, at least in part, for the reported beneficial effects of this peptide in DKD.

In cultured podocytes, we showed that high glucose downregulates *APJ* mRNA levels and this effect is markedly accentuated by exposure to Ang II (Fig. 8). These findings prompted us to study the effect of diabetes in vivo on kidney APJ expression in the *db/db* mouse model of DKD. In these animals, urinary albumin excretion was increased as previously reported (Hummel et al. 1966; Sharma et al. 2003) and glomerular mesangial expansion was present as well as an increase in the glomerular tuft area in glomeruli from both cortex and juxtamedullary region. A causative role of suppressed APJ expression in the development of DKD requires further evidence but several findings suggest its important role. The proapoptotic action of high glucose, as inferred by Caspase‐3 activity, was suppressed by simulation of APJ with apelin‐13 in cultured podocytes. We also showed that Ang II downregulates both *APJ* and *preproapelin* in podocytes exposed to high glucose. In the *db/db* model, like in other models of DKD in mice and humans (Tamura et al. 2005; Peti‐Peterdi et al. 2008; Umemoto et al. 2016; Wysocki et al. 2017a,b), the kidney RAS is overactive as reflected by high levels of urinary angiotensinogen and Ang II. This upregulation is at least partially attributable to stimulation by the high glucose environment (Wolf 2004; Peti‐Peterdi et al. 2008). Overall, our data suggest that high glucose and high Ang II activity may converge to suppress APJ in podocytes and contribute to the development of the glomerular lesions of DKD and its progression.

The suppression of *APJ* and *preproapelin* that we found in podocytes was previously described in different tissues such as adipocytes and cardiomyocytes, which was reported to be dose‐dependent and mediated by the AT1‐receptor (Iwanaga et al. 2006; Wu et al. 2014). The combination of high glucose and high levels of Ang II achieved a higher level of *APJ* and *preproapelin* downregulation than the high glucose environment alone (Fig. 8). This synergistic effect may explain the low level of APJ observed in the *db/db* mouse model of DKD where we found that the RAS is overactive at the kidney level (i.e., increased urinary AOG and Ang II). Overactivity of the kidney RAS is a feature of other models of DKD and human DKD (Tamura et al. 2005; Peti‐Peterdi et al. 2008; Umemoto et al. 2016; Wysocki et al. 2017a,b; Patney et al. 2018). Possibly downregulation of the kidney APJ is, at least in part, the consequence of hyperglycemia and RAS overactivity at the tissue level.

Of interest, specific AT1 blockade with telmisartan resulted in an increase in the *APJ* and *preproapelin* mRNA in *db/db* mice. Upregulation of these two major components of the apelinergic system, *APJ* and *preproapelin*, may be yet another beneficial effect of AT1‐blockers beyond AT1‐inhibition.

In conclusion, the present study shows that in the kidney glomerulus the apelin receptor is preferentially localized in podocytes. In cultured podocytes, high glucose and Ang II decrease *APJ* expression at the mRNA level while the apoptotic effect of high glucose can be reversed by stimulation of APJ by one of its main ligands Apelin‐13. In diabetic *db/db* mice kidney, APJ expression is markedly decreased at the mRNA level and protein level. Altogether our findings suggest a role for APJ downregulation in the development of DKD. The half‐life of apelin‐13 and other apelins is short (minutes) but with the development of apelin agonists with prolonged half‐life (Juhl et al. 2016; Huang et al. 2018), the podocyte apelinergic system may become a good target for the treatment of DKD.

## Conflict of Interest

None declared.
